# Systemic Inflammation in Oncologic Patients Undergoing Systemic Treatment and Receiving Whey Protein-Based Nutritional Support

**DOI:** 10.3390/ijms25115821

**Published:** 2024-05-27

**Authors:** Aura D. Herrera-Martínez, Ana Navas Romo, Soraya León-Idougourram, Concepción Muñoz-Jiménez, Rosa Rodríguez-Alonso, Gregorio Manzano García, Marta Camacho-Cardenosa, Antonio Casado-Diaz, María Ángeles Gálvez-Moreno, María José Molina Puertas, Aurora Jurado Roger

**Affiliations:** 1 Maimonides Institute for Biomedical Research of Cordoba (IMIBIC), 14004 Córdoba, Spainsorayaleon3@hotmail.com (S.L.-I.); carri2976@gmail.com (C.M.-J.); marta.camacho@imibic.org (M.C.-C.); cmmerinomjmolina@hotmail.com (M.J.M.P.); 2 Endocrinology and Nutrition Service, Reina Sofia University Hospital, 14004 Córdoba, Spain; 3 Clinical Immunology Service, Reina Sofia University Hospital, 14004 Córdoba, Spain; 4 Medical Oncology Service, Reina Sofia University Hospital, 14004 Córdoba, Spain; 5 CIBER Fragilidad y Envejecimiento Saludable (CIBERFES), Instituto de Salud Carlos III, 08003 Madrid, Spain

**Keywords:** inflammation, cytokines, whey protein, cancer

## Abstract

There is increasing evidence about the role of inflammation in sarcopenia and tumor progression; thus, its modulation would represent a valuable strategy for improving clinical outcomes in patients with cancer. Several studies have reported that whey protein has significant anti-inflammatory and antioxidant characteristics in humans. We aimed to evaluate the effects of whey protein-based oral nutritional support on circulating cytokines in patients with solid tumors undergoing systemic treatment. Forty-six patients with solid tumors of different origin and undergoing systemic treatment were evaluated. Nutritional support with two daily whey protein-based oral supplements was administered. Circulating levels of IL-6, IL-8, IL-10, MCP-1 and IP-10 were determined. Nutritional evaluation included anthropometric, instrumental and biochemical parameters. Over 63% of the evaluated patients underwent surgery, 56.5% required chemotherapy and almost 50% received combined treatment. Patients with resected primary tumor presented with lower baseline IL-6 (*p* < 0.05) and IP-10 (*p* < 0.001); after three months of nutritional support, they presented with lower IL-8 (*p* < 0.05) and tended to present lower IL-6 and IP-10 (*p* = 0.053 and 0.067, respectively). Significant positive correlations between circulating cytokines, C-reactive protein and ferritin were observed; similarly, negative correlations with anthropometric and biochemical nutritional parameters were noticed (*p* < 0.05). We did not observe significant changes in circulating cytokine levels (IL-6, IL-8, IL-10, MCP-1 and IP-10) in patients with cancer undergoing systemic treatment after three months of nutritional support with whey protein-based oral supplements. According to a univariate analysis in our cohort, circulating IL-8 was associated with mortality in these patients, additionally, MCP-1 and IP-10 tended to correlate; but an age- and sex-adjusted multivariate analysis revealed that only baseline MCP-1 was significantly associated with mortality (OR 1.03 (95% CI: 1.00–1.05)). In conclusion, surgery of the primary solid tumor and combination treatment allow significant reduction in circulating cytokine levels, which remained stable while patients received nutritional support with whey protein-based oral supplements over three months. The role of MCP-1 as an independent factor for mortality in these patients should be further evaluated.

## 1. Introduction

There is growing evidence that suggests that chronic inflammation promotes carcinogenesis and may increase the risk of certain cancer types, including bladder, liver, colorectal, lung, esophagogastric junction and stomach [1,2]. Several mechanisms have been suggested, including continual unregulated proliferation, apoptosis and genetic mutations [3], as well as the release of several proinflammatory cytokines associated with reactive oxygen or nitrogen species, which have been widely associated with tumor promotion and progression [4]. Additionally, cancer itself also promotes inflammation [5], which generates a continuous inflamed condition, which is difficult to control.

Evidence also supports the effect of chronic inflammation on sarcopenia and muscle loss, which are independently associated with increased morbidity and mortality, especially in cancer patients [6]. Furthermore, inflammation could be also increased due to the clinical effect of some treatments including chemo- and radiotherapy in these patients [7,8].

Currently, new possibilities for the creation of interventions related with metabolism and inflammation are matters of interest. Specifically, increased anti-inflammatory nutritional intake seems to be associated with lower risk of overall mortality including metabolic disorders, sarcopenia and cancer [9]. In this context, the design of nutritional intervention strategies that improve the inflammatory phenotype of some patients would help to improve the clinical outcome of certain diseases [10].

Previous studies have reported that antioxidant-rich diets have an inverse association with the risk of certain types of cancer, since antioxidants prevent free radical-induced oxidative damage to cells, allowing the neutralization of reactive oxygen species [11]. In this context, whey protein could represent a dietary modulator of inflammation, since it possesses both antioxidant and anti- inflammatory characteristics as one of the richest sources of branched-chain amino acids like leucine; additionally, according to some studies, the whey proteins lactoferrin and α-lactalbumin and the κ-casein-derived peptide glycomacropeptide have various bioactivities, including immunomodulatory properties [12,13]. Moreover, as whey protein is digested, it releases an array of peptides, which also have an immunomodulatory function [14].

Several studies have suggested that whey protein can modulate immune function in older adults; furthermore, cytokine reductions have been also reported, especially in subjects older than 50 years old, suggesting that whey protein could play a role in preventing and treating sarcopenia [15]. Moreover, some authors have suggested that whey protein can also trigger apoptosis, hinder tumor cell proliferation, and impede metastasis [16]; thus, an additional effect on tumor response to systemic treatment in cancer patients could be expected.

In this context, this study aimed to evaluate the circulating levels of some sarcopenia-related cytokines in patients with solid tumors undergoing systemic treatment (with chemotherapy, radiotherapy or a combination of them) and determine their evolution after twelve weeks (three months) of nutritional support with whey protein-based oral supplements.

## 2. Results

### 2.1. Baseline Characteristics of the Patients

Forty-six patients were evaluated. The majority was female (54.3%), and the median age was 74 years old. Primary tumor sites included colon (19.6%), urothelium (32.6%), head–neck cancer (13%), gastric cancer (8.7%), gastrointestinal neuroendocrine tumor (8.7%) and other localizations (17.4%). Over 63% of the evaluated patients underwent surgery, 56.5% required chemotherapy and almost 50% received combined treatment; this last group included surgery in combination with chemotherapy, radiotherapy or a combination of the last two. Weight loss during the previous three months was a very common symptom (over 84%) compared with weight loss over a longer period of time; almost half of the patients presented with gastrointestinal symptoms and any level of dependency, but ECOG 0 was observed in up to 60% of the evaluated patients (Table 1).

Patients with previous history of other neoplasms tended to present with increased circulating IL-6 levels ((median 3.2 pg/mL (IQR 74.3) vs. 0 pg/mL (IQR 2.90), *p* = 0.06). No significant differences in baseline circulating cytokine levels were found for sex, previous history of type 2 diabetes or tobacco exposure.

**Figure 1:**
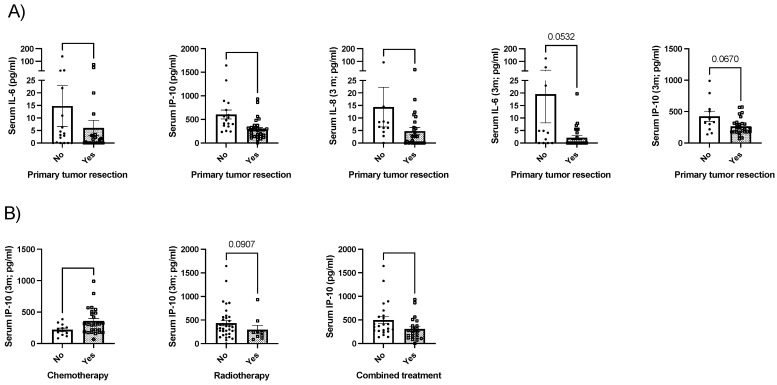
Clinical associations between cytokine levels and (**A**) surgery; (**B**) other therapies including chemotherapy, radiotherapy or combined treatment. Legend: *: *p* < 0.05; *** *p* < 0.001; serum cytokine levels are expressed in pg/mL.

Patients with resected primary tumor presented with lower baseline IL-6 (*p* < 0.05) and IP-10 (*p* < 0.001); after three months of nutritional support, they presented with lower IL-8 (*p* < 0.05) and tended to present lower IL-6 and IP-10 (*p* = 0.053 and 0.067, respectively; Figure 1A). Any other significant association was observed when the other cytokines were analyzed.

Patients that underwent chemotherapy presented with increased IP-10 levels after three months (*p* < 0.05); in contrast, IP-10 tended to be decreased in patients that underwent radiotherapy (*p* = 0.09). Moreover, patients that required combined treatment (surgery plus chemotherapy, radiotherapy or both) presented with lower baseline IP-10 levels (*p* < 0.05). Any other significant association was observed when the other cytokines were analyzed.

### 2.2. Clinical Correlations between Cytokines and Morphofunctional Nutritional Evaluation in Cancer Patients

**Figure 2:**
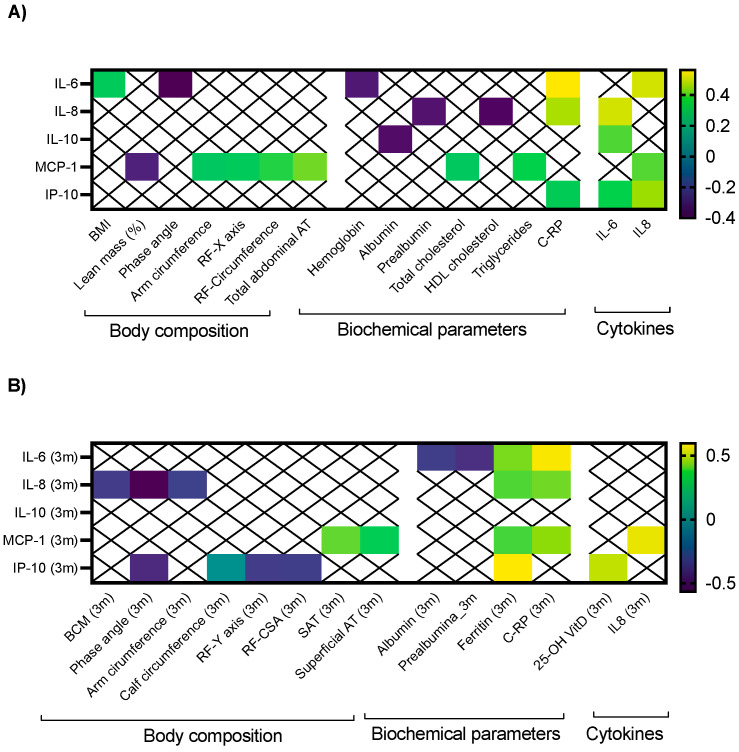
Significant clinical correlations between nutritional parameters and (**A**) baseline cytokine levels; (**B**) circulating cytokines after a nutritional intervention of three months in patients with cancer receiving systemic treatment. Legend: only significant correlations are depicted. Color boxes represent the strength of the correlation according to the color scale represented on the right side of each figure.

Baseline IL-6 presented a positive correlation with BMI (r: 0.323), CRP (r: 0.562) and IL-8 (0.502); in contrast, it negatively correlated with phase angle (r: −0.403) and hemoglobin (r: −0.326; Figure 2A). After three months of nutritional support, no significant correlation with body composition parameters was observed. Regarding biochemical parameters, post-treatment IL-6 levels negatively correlated with albumin, prealbumin and positively correlated with ferritin and C-RP (r: 0.422 and 0.575, respectively; Figure 2B).

Baseline IL-8 also positively correlated with C-RP and negatively with prealbumin and HDL cholesterol (Figure 2A). Three months later, it negatively correlated with BCM, phase angle and arm circumference, while it positively correlated with ferritin and C-RP (Figure 2B).

IL-10 negatively correlated with albumin (r: −0.357) and circulating IL-6 at baseline (Figure 2A). During follow-up, any significant correlation was observed with body composition or biochemical parameters (Figure 2B).

Baseline MCP-1 negatively correlated with lean mass, and positively with RF-transversal axis, RF-circumference, arm circumference and total abdominal adipose tissue. Regarding biochemical parameters, it positively correlated with total cholesterol, triglycerides and IL-8. After three months of nutritional intervention, it still positively correlated with subcutaneous adipose tissue, superficial abdominal adipose tissue, ferritin, C-RP and IL-8.

Finally, baseline IP-10 correlated in a positive manner with C-RP, IL-6 and IL-8 (Figure 2A). After three months, significant negative correlations with phase angle, RF-Y axis and RF-cross sectional area were observed; in contrast, positive correlations with ferritin and 25-OH vitamin D were also noticed (Figure 2B).

### 2.3. Whey Protein-Based Nutritional Support and Clinical Outcomes

After three months of nutritional support, any significant difference was observed in body composition parameters determined via BIA, anthropometric parameters (calf, arm and abdominal circumferences), handgrip strength and RF-muscle ultrasound. In the abdominal ultrasound, superficial subcutaneous adipose tissue increased from 0.51mm (0.37–0.68) to 0.59 (0.36–0.8; *p* = 0.02) and the stand-up test improved from 9 repetitions per minute (0–17) to 10 (4–22; *p* < 0.03). Regarding biochemical parameters, prealbumin increased from 23 mg/dL (8.9–56) to 24 mg/dL (5–36; *p* = 0.02), C-RP significantly decreased (5.1 2.8 g/dL (0.5–10309) at baseline to 2.8 g/dL (0–147) after three months (*p* = 0.03)) and 25-OH vitamin D increased from 12 ng/dL (7–30) to 33 ng/dL (16–68; *p* < 0.001). Any other significant changes in biochemical nutrition-related parameters were observed.

### 2.4. Whey Protein-Based Nutritional Support and Circulating Cytokine Levels

**Figure 3:**
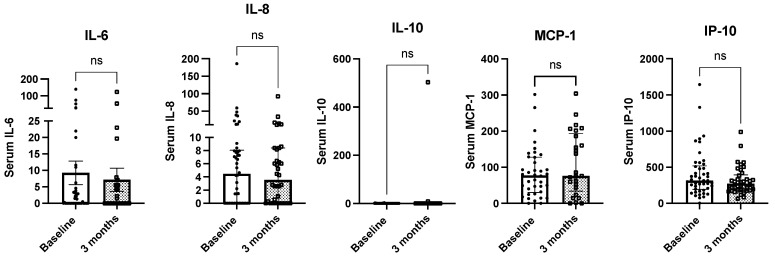
Changes in circulating serum cytokine levels after three months of nutritional intervention in patients with cancer undergoing systemic treatment. Legend: ns: non-significant.

**Figure 4:**
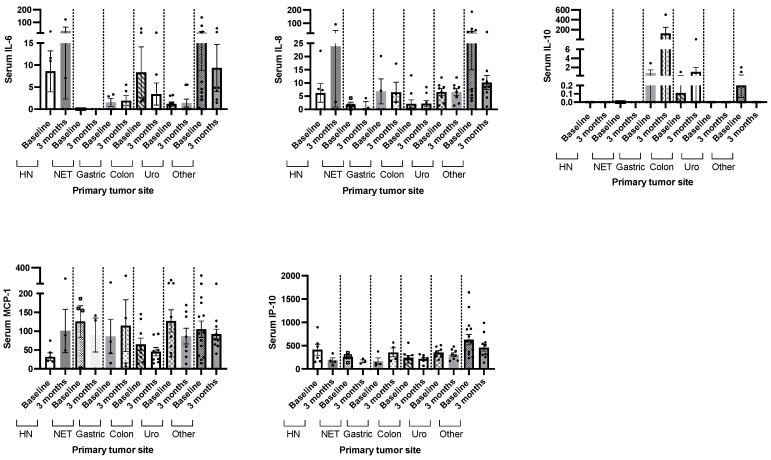
Changes in circulating serum cytokine levels after three months of nutritional intervention in patients with cancer undergoing systemic treatment divided by primary tumor site. Legend: HN: head–neck cancer; NET: neuroendocrine tumor of the gastrointestinal tract; Uro: urothelial.

We did not observe significant changes in circulating cytokine levels (IL-6, IL-8, IL-10, MCP-1 and IP-10) in patients with cancer undergoing systemic treatment after three months of nutritional support with whey protein-based oral nutritional supplements (Figure 3). Significant differences in the circulating levels of serum IL-6, IL-8, IL-10, MCP-1 and IP-10 after three months of the nutritional intervention were observed in patients with head and neck cancer, neuroendocrine tumors, gastric, colon, urothelial or other types of cancer (Figure 4).

**Figure 5:**
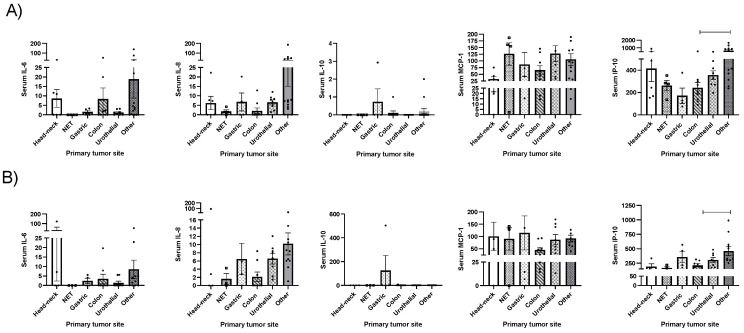
Primary cancer type and circulating cytokine levels at (**A**) baseline and (**B**) after three months of treatment. Legend: * *p* < 0.05.

Additionally, we did not observe significant differences in circulating cytokine levels when primary cancer type was compared, but for IP-10 levels, which were lower in colon cancer compared with other types of cancer at baseline and after three months (Figure 5).

### 2.5. Mortality and Circulating Cytokine Levels

**Figure 6:**
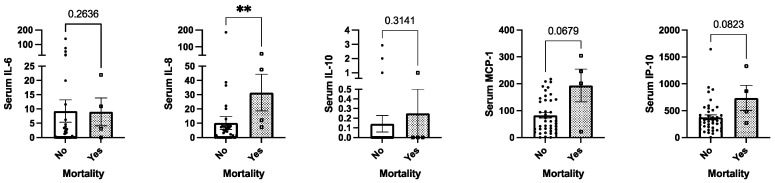
Circulating serum cytokin levels and mortality in patients with cancer undergoing systemic treatment (univariate analysis). Legend: ** *p* < 0.01.

According to a univariate analysis in our cohort, circulating IL-8 was associated with mortality in these patients (*p* < 0.05); also, MCP-1 and IP-10 tended to correlate (*p* = 0.06, *p* = 0.08, respectively; Figure 6). Nevertheless, after age- and sex-adjusted analysis, only baseline MCP-1 was significantly associated with mortality (OR 1.03 (95% CI: 1.001–1.05), Table 2).

## 3. Discussion

There is growing evidence about the significant role of chronic inflammation in the clinical course of several diseases, including cancer and sarcopenia. Furthermore, patients with cancer (and specially with solid tumors) present with significant weight loss, malnutrition and sarcopenia, which worsens their tumor-related inflammatory status [17]. In this context, it is mandatory to develop therapeutic strategies that could trigger this inflammatory condition to improve the clinical prognosis and the quality of life of these patients.

Cancer cachexia is a multifactorial condition, frequently observed in patients with solid tumors, that affects clinical outcomes and prognosis. It is a consequence of decreased nutrient accessibility (due to anorexia or malabsorption) and metabolic abnormalities, triggered by a complex network of cytokines, hormones and other tumor- and host-derived humoral factors [18]. Additionally, anti-neoplastic therapies are accompanied by different side effects that can also exacerbate this condition [19]. Frequently, cancer cachexia is accompanied by muscle wasting, which is considered a prognostic factor for cancer patients, since it is associated with higher incidence of chemotherapy toxicity, shorter time to tumor progression, poorer surgical outcome, physical impairment and shorter survival [20,21].

Surgical resection remains the only curative treatment for several solid cancers, especially for localized disease. However, some studies have reported that after surgery, a transient postoperative immunosuppression occurs, which provides a window for cancer cell proliferation leading to rapid recurrences or metastases. This immunosuppressive status after surgery seems to be associated with the severity of surgical trauma, since the systemic response to tissue damages and wound healing induce cellular immunity activation [22]. In contrast, in our cohort, we observed that patients with resected primary tumors presented with reduced circulating cytokine levels. These differences might be related to the type of measured cytokine, since after surgery, interferon-γ (IFN-γ) is more affected; this cytokine has been related to tumor progression and metastasis and was not evaluated in this study, since we focused on sarcopenia or nutrition-related cytokines. Additionally, the type of surgery and the time that has elapsed since the intervention also influence the systemic inflammatory status [22,23], which is lower with minimally invasive techniques, and increased in major surgery [24] or in the period immediately following the procedure [25,26]. On the other hand, it is reasonable to think that the elimination of the tumor mass decreases the proinflammatory state. In contrast, and as observed in our cohort, chemotherapy has been widely associated with tumor cell damage and consequent increased inflammation, which has been associated in some cases with worse treatment tolerance and effectiveness [27,28].

Among the evaluated cytokines, IL-6 has been widely associated with obesity and increased metabolic risk [29], which is reflected in our cohort with its positive correlation with BMI and negative correlation with phase angle. IL-6 is also one of the major cytokines in the tumor microenvironment; its overexpression has been reported in almost all types of tumors [30]. To the best of our knowledge, nutritional compounds are currently under study in humans for modulating this signaling pathway; recently, a study has described the potential use of the fruit peel of Moringa oleífera for regulating cytokine levels of hepatocarcinoma in an animal model [31]. Additionally, its modulation using tocilizumab in inflammatory diseases and canakinumab in patients with stable atherosclerosis has been previously reported [32,33] and several other modulating agents are under study [33].

IL-8 favors the transition of tumor cells to a mesenchymal phenotype, increases migration and promotes cell proliferation in cancer [34]. Furthermore, in pancreatic tumors, it is an independent marker of survival and is strongly correlated with cancer cachexia, weight loss and sarcopenia [35]. After three months of nutritional intervention, it negatively correlated with BCM, phase angle and arm circumference, similar to other studies in which it has been negatively correlated with muscle mass-related parameters, including psoas muscle area [35]. Previous studies have reported that consumption of food with increased sugar-free content produces increased serum levels of IL-8 [36]; additionally, the fact that after nutritional support this cytokine negatively correlated with muscle mass-related markers but positively with ferritin and C-RP suggests that it might be modulated after nutritional support in patients with cancer, but its effect should be assessed after longer interventions.

In our cohort, we observed that MCP-1 was associated with adipose tissue and metabolic markers, as previously reported [37]; moreover, it has been associated with fat mass and consumption of high-fat diets [38,39]. Other studies have suggested that MCP-1 has also a key role in angiogenesis, tumor invasion capacity, metastasis and immune cell infiltration in solid tumors [40]. Its clinical relation with increased mortality has been reported in different scenarios, including critic patients, sepsis and coronary disease [41,42,43]. In this context, its modulation has been tested in animal models with poor results [44], but the clear association with mortality observed in our study suggests that that should be further evaluated as a putative therapeutic marker that could improve mortality rates.

Finally, IP-10 seems to promote tumor growth, migration and invasion of cancer cells in several tumor types [45], while in sarcopenia it has been described as a marker of physical frailty and sarcopenia [46]. It is interesting that after nutritional support, it negatively correlated with lean mass-related parameters, suggesting an inverse relation in muscle mass gain, thus a potential value as a marker for improved muscle mass function.

Circulating cytokine level modulation using diet interventions has been suggested in different clinical scenarios; for example, some studies have reported decreased IL-6 levels after the consumption of low-calorie diet, berry, whole wheat and polyunsaturated fatty acids in patients with metabolic syndrome [47], decreased IL-6 levels after supplementation with branched-chain amino acids, calcium and vitamin D3 in patients with elderly sarcopenia [48], or decreased IL-18 levels after Mediterranean diet and supplementation with very long chain omega-3 polyunsaturated fatty acid in men with high-risk of atherosclerosis (despite mild changes in body composition parameters) [49]. In contrast, in this study we did not observe significant changes in circulating cytokine levels after three months of nutritional support; far from considering this fact as a treatment failure, it represents a positive clinical response to nutritional treatment, since all patients were receiving systemic treatment during the study period, and despite this, circulating cytokine levels did not increase, as could be expected. Furthermore, the association between cytokine levels and mortality in patients with solid tumors reflects the significant effect of nutritional support in preventing mortality in these patients. Unfortunately, this study did not evaluate mechanistic pathways that could have been implicated, but future study on the matter should be performed.

This study has some limitations, specially related with the different primary tumor locations, which implies different surgical approaches and different systemic treatment; however, as an exploratory study, it is relevant and provides a basis for further work with homogeneous pathological groups. This limitation was managed when cytokine levels were analyzed at baseline comparing different tumor sites, since we observed that circulating levels were comparable in the evaluated solid tumors. Another limitation has been already mentioned: the evaluation of mechanistic pathways (including mRNA cytokine expression, CD4/CD8 circulating and tumor levels, among others) could provide additional, valuable information about real nutritional-related modulation. Ideally, a comparison with a placebo would reflect the specificity of the nutritional intervention on systemic treatment, but it is not ethically correct to deny nutritional support to patients with positive nutritional screening; this is a frequent limitation in studies that evaluate nutritional interventions [50]. Finally, a longer period of evaluation could provide additional information about long-term support. In contrast, this study has several strengths, all included patients presented with malnutrition with an active cancer receiving systemic treatment, which is a reflection of the reality in clinical practice; furthermore, only patients with a high level of adherence to nutritional support were included, and finally a comprehensive nutritional evaluation was performed in all cases.

Altogether, our results reveal that surgery and systemic treatment influences circulating cytokines in patients with solid tumors and are closely related with tumor prognosis and clinical evolution. Whey protein-based nutritional support results in maintenance of circulating levels of cytokines despite patients receiv active systemic treatment, suggesting a positive effect on non-tumoral cell mass maintenance. Its effect as an isolated tool for triggering inflammation in cancer patients should be confirmed in larger cohorts during longer interventions and reinforced with molecular studies.

## 4. Material and Methods

### 4.1. Patients

This study was approved by the Ethics Committee of the Reina Sofia University Hospital (Cordoba, Spain; reference number 4788, approved on 30 October 2020). It was conducted in accordance with the Declaration of Helsinki and according to national and international guidelines. All patients received information before the inclusion and if accepted to participate, a written informed consent was signed by every individual. In this study, patients of both sexes, age > 18 years old, with solid tumors undergoing systemic treatment (chemotherapy, radiotherapy or combination treatment) that presented with weight loss > 5% during the previous three months or >10% during the previous six months were included, as recognized according to different nutritional screening tools [51,52]. In our center, it is protocol that patients with these characteristics are derived for nutritional evaluation and support before starting systemic treatment, for improving clinical outcomes and treatment tolerance [53]. This study reflects the current clinical practice in this outpatient clinic. Medical history data were collected, including epidemiological information, primary cancer type, type of systemic treatment (including surgery, chemotherapy, radiotherapy or their combination), nutrition-related symptoms and quality of life (which was determined using a 0 to 100 self-reported scale) and the Eastern Cooperative Oncology Group (ECOG) scale. Any level of dependency (for routine activities including feeding, dressing, bath or walking) was also determined. The primary tumor was treated according to current clinical guidelines.

### 4.2. Study Protocol, Chronogram and Nutritional Support

Forty-six consecutive patients with cancer of different origins (solid tumors) on systemic treatment (chemotherapy, radiotherapy or combination treatment) were included. Exclusion criteria included end-stage kidney or liver function. Any patient with pregnancy or mental or cognitive problems was included. All patients received nutritional support with two hypercaloric, hyperproteic, whey protein-based oral nutritional supplements (ONS) over twelve weeks (hereafter three months). Nutritional supplements had a caloric density of 1.51–1.6 Kcal/mL, with 1.51–1.6 g of proteins, 14.8–15.7 g of carbohydrates and 5.2–6.7 g of fat per 100 mL of product. Each bottle contained 200 mL of product; two bottles per day were prescribed; patients are advised to drink both ONS in small quantities fractioned along the whole day, to not reduce their regular food intake. All patients also received general education and advice about nutritional support, Mediterranean diet and physical activity; additionally, oral supplementation with calcifediol was also administered (in different doses in order to reach levels of sufficiency, defined with a serum 25OH vitamin D levels > 30 ng/dL). Any patient received a placebo; due to ethical reasons, all patients received nutritional support. All patients were evaluated at baseline before starting systemic treatment with chemo- or radiotherapy (as individually required according to the primary tumor location, stage and clinical prognosis); nutritional support was started and patients were evaluated three months after they started the nutritional support; only patients that presented an adherence to the nutritional supplement > 75% were included in the study.

### 4.3. Nutritional and Anthropometric Evaluation

A morphofunctional nutritional evaluation was performed as previously described [6,17,54]. Briefly, physical examination included body composition analysis (bioelectrical bioimpedance, abdominal, arm and calf circumferences), functional tests (up and go test and handgrip strength) and nutritional ultrasound of abdominal adipose tissue and rectus-femoris muscle of the quadriceps. Specifically, vectorial bioelectrical bioimpedance (BIVA) was performed using a NUTRILAB-Akern impedanciometer (Florence, Italy); this study reported body composition parameters including fat mass, lean mass, water, bone, phase angle, body cell mass (BCME) and extracellular mass (ECME). Nutritional evaluation also included anthropometric parameters (calf, arm and abdominal circumference). For handgrip strength, a Jamar^®^ hydraulic dynamometer (Asimow Engineering Co., Los Angeles, CA, USA was used. A stand-up test was also performed (number of times that the patients stand up from a seated position in one minute). Nutritional ultrasound was performed using a GE Logiq E9 ultrasound machine and a linear 9L-D probe (Madrid, Spain); specifically, two ultrasounds were performed: (1) abdominal adipose tissue (AT) ultrasound, in which total abdominal adipose tissue, subcutaneous adipose tissue and pre-peritoneal fat were determined, and (2) recuts femoris (RF) ultrasound, in which subcutaneous AT, RF-Y (anteroposterior) and RF-X (transversal) axis, muscle area and circumference were determined. The presence of malnutrition according to the GLIM criteria [55] and sarcopenia (defined as an age- and gender-adjusted handgrip strength ≤ p25) was also determined. Biochemical nutritional analyses were also performed (hemoglobin, lymphocytes, total cholesterol, total high-density lipoprotein (HDL) cholesterol, low density lipoprotein (LDL) cholesterol, triglycerides, transferrin, albumin, prealbumin) and inflammation markers as C-reactive protein (C-RP) and ferritin were included.

### 4.4. Cytokine and Chemokine Measurement

Blood samples were collected the same day of the nutritional evaluation. Cytokines and chemokines were measured in serum; all samples were stored at −80° until their simultaneous measurement under the same environment conditions. Cytokines were quantified via Cytometry Bead Array (CBA, BD Cytometric Bead Array Human Soluble Protein Master, ref. 558264/558265; Becton Dickinson and Company, San Jose, CA, USA). The following cytokines were analyzed according to the manufacturer’s instructions: IL-6 (ref. 558276), IL-8 (CXCL8, ref. 558277), IL-10 (ref. 558274), MCP-1 (CCL2, ref. 558287) and IP-10 (CXCL10, ref. 558280). For sample acquisition, a FACS Canto II was used, and a minimum of 300 events were recorded per each cytokine. Median Fluorescence Intensity (MFI) data were transformed in concentration (pg/mL) using a calibration curve as a reference. These cytokines were selected for evaluation based on literature reports [30,34,37].

### 4.5. Statistical Analysis

The Kolmogorov–Smirnov test was used to assess the normal distribution of data. For the descriptive statistics, the mean, median, standard deviation and interquartile range of the continuous variables and the frequencies and percentages of the discrete variables were calculated. To assess differences between the continuous variables, the Mann–Whitney U test was used (nonparametric data). Paired analysis was performed via the Wilcoxon test (nonparametric data). For differences between the discrete variables, Pearson´s test was used (clinical correlations). The odds ratio (OR) and 95% confidence intervals (CIs) were obtained using logistic regression analysis. Statistical analyses were performed using SPSS statistical software version 20, and Graph Pad Prism version 6. Significance was defined as a *p*-value of <0.05.

## Figures and Tables

**Table 1:** Baseline clinical characteristics of the patients.

Characteristics	Total (*n* = 46)
Sex (♂/♀)	45.7/54.3% (21/25)
Age at diagnosis (years)	74.5 (71–78)
**Tobacco exposure**	
No	50% (21/42)
Active	28.6% (12/42)
Previous exposure	21.4% (9/42)
**Previous other neoplasms**	19.7% (9/46)
**Primary tumor location**	
Head/neck	13% (6/46)
Gastrointestinal NET	8.7% (4/46)
Gastric cancer	8.7% (4/46)
Colorectal cancer	19.6% (9/46)
Urothelial	32.6% (15/46)
Others	17.4% (8/46)
**Treatment**	
Surgery	63% (29/46)
Chemotherapy	56.5% (26/46)
Radiotherapy	19.6% (9/46)
Chemo and radiotherapy	23.9% (11/46)
Combined treatment (surgery plus systemic treatment)	47.8% (22/46)
**Symptoms**	
Weight loss (3 months)	84.4% (38/45)
Weight loss in Kg (3 months)	5 (4–6)
Weight loss (6 months)	71.7% (33/46)
Weight loss in Kg (6 months)	6.5 (6–7)
Gastrointestinal symptoms	43.5% (20/46)
Abdominal pain	32.6% (15/46)
Nauseas/vomits	22.2% (10/45)
Diarrhea	15.2% (7/46)
Dyspnea	17.4% (8/46)
Mucositis	8.7% (4/46)
**Quality of life**	
Any level of dependency	43.5% (20/46)
Self-rated health score	65 (0–80)
ECOG	
ECOG 0	60.9% (28/46)
ECOG 1	28.3% (13/46)
ECOG 2	10.9% (5/46)
**Mortality**	8.7% (4/46)

Legend: NET: neuroendocrine tumor; ECOG: Eastern Cooperative Oncology Group performance scale.

**Table 2:** Multivariate logistic regression for mortality in patients with cancer undergoing systemic treatment after adjusting by age and sex.

Variable		OR	CI	*p*
Mortality	Baseline IL-6	0.98	0.94–1.03	0.62
	Baseline IL-8	1.02	0.99–1.04	0.18
	Baseline IL-10	1.32	0.21–8.00	0.76
	Baseline MCP-1	1.03	1.001–1.05	0.03
	Baseline IP-10	1.00	1.00–1.01	0.08

## Data Availability

The original contributions presented in the study are included in the article, further inquiries can be directed to the corresponding authors.
